# Protease-anti-protease compartmentalization in SARS-CoV-2 ARDS: Therapeutic implications

**DOI:** 10.1016/j.ebiom.2022.103894

**Published:** 2022-02-22

**Authors:** Oisin F. McElvaney, Takanori Asakura, Suzanne L. Meinig, Jose L. Torres-Castillo, Robert S. Hagan, Claudie Gabillard, Mark P. Murphy, Leigh B. Thorne, Alain Borczuk, Emer P. Reeves, Ross E. Zumwalt, Yu Mikami, Tomas P. Carroll, Kenichi Okuda, Grace Hogan, Oliver J. McElvaney, Jennifer Clarke, Natalie L. McEvoy, Patrick W. Mallon, Cormac McCarthy, Ger Curley, Matthew C. Wolfgang, Richard C. Boucher, Noel G. McElvaney

**Affiliations:** a Irish Centre for Genetic Lung Disease, RCSI Education and Research Centre, Beaumont Hospital, Dublin 9, Dublin, Ireland; b Marsico Lung Institute/Cystic Fibrosis Research Center, University of North Carolina at Chapel Hill, Chapel Hill, NC, USA; c Department of Anaesthesia and Critical Care, Beaumont Hospital, Dublin, Ireland; d Royal College of Surgeons in Ireland, Dublin, Ireland; e Alpha-1 Foundation, Ireland; f Division of Pulmonary Diseases and Critical Care Medicine, Department of Medicine, University of North Carolina, Chapel Hill, NC 27599, USA; g Department of Pathology and Laboratory Medicine, University of North Carolina at Chapel Hill, Chapel Hill, NC, USA; h Department of Pathology and Laboratory Medicine, Weill Cornell Medicine, New York, NY, USA; i Department of Pathology, University of New Mexico School of Medicine, Albuquerque, NM, USA; j Department of Microbiology and Immunology, University of North Carolina School of Medicine, Chapel Hill, NC 27599, USA; k Department of Infectious Diseases, St Vincent's University Hospital, Dublin, Ireland; l Centre for Experimental Pathogen Host Research (CEPHR), University College Dublin, Dublin, Ireland; m Department of Respiratory Medicine, St Vincent's University Hospital, Dublin, Ireland; n School of Medicine, University College Dublin, Dublin, Ireland

**Keywords:** Alpha-1 antitrypsin, SARS-CoV-2 infection, Neutrophil elastase, Interleukin-6

## Abstract

**Background:**

Interleukin-6 (IL-6) is elevated in SARS-CoV-2 infection. IL-6 regulates acute-phase proteins, such as alpha-1 antitrypsin (AAT), a key lung anti-protease. We investigated the protease-anti-protease balance in the circulation and pulmonary compartments in SARS-CoV-2 acute respiratory distress syndrome (ARDS) compared to non-SARS-CoV-2 ARDS (nsARDS) and the effects of tocilizumab (IL-6 receptor antagonist) on anti-protease defence in SARS-CoV-2 infection.

**Methods:**

Levels and activity of AAT and neutrophil elastase (NE) were measured in plasma, airway tissue and tracheal secretions (TA) of people with SARS-CoV-2 ARDS or nsARDS. AAT and IL-6 levels were evaluated in people with moderate SARS-CoV-2 infection who received standard of care +/- tocilizumab.

**Findings:**

AAT plasma levels doubled in SARS-CoV-2 ARDS. In lung parenchyma AAT levels were increased, as was the percentage of neutrophils involved in NET formation. A protease-anti-protease imbalance was detected in TA with active NE and no active AAT. The airway anti-protease, secretory leukoprotease inhibitor was decreased in SARS-CoV-2-infected lungs and cleaved in TA. In nsARDS, plasma AAT levels were elevated but TA samples had less AAT cleavage, with no detectable active NE in most samples

Induction of AAT in ARDS occurred mainly through IL-6. Tocilizumab down-regulated AAT during SARS-CoV-2 infection.

**Interpretation:**

There is a protease-anti-protease imbalance in the airways of SARS-CoV-2-ARDS patients. This imbalance is a target for anti-protease therapy.

**Research in context**

**Evidence before this study**

Severe acute respiratory syndrome coronavirus 2 (SARS-CoV-2) infection is characterised by an IL-6 driven cytokinemia, associated with a rapidly developing acute respiratory distress syndrome (ARDS). A blunted AAT response to IL-6 in SARS-CoV-2 has been associated with increased morbidity and mortality.

**Added value of this study**

This study shows that the AAT response to SARS-CoV-2 infection is compartmentalized with an appropriate increase in plasma and alveoli but an inadequate response in airways. This underlines a significant, but potentially treatable, protease-antiprotease imbalance in SARS-CoV-2 ARDS as well as highlighting IL-6’s importance in SARS-CoV-2 pathology not only as a pro-inflammatory cytokine but as an anti-inflammatory regulator.

**Implications of all the available evidence**

There is unopposed NE activity in the airways of people with SARS-CoV-2 ARDS, which could be amenable to AAT therapy. Our data suggest caution in the use of IL-6 blocking therapies in SARS-CoV-2-infected individuals.

Alt-text: Unlabelled box

## Introduction

Severe acute respiratory syndrome coronavirus 2 (SARS-CoV-2) infection is a major global health risk with few therapies available to modify its clinical course.^1^ As of September 2021, more than 228 million laboratory-confirmed cases have been documented globally, with over 4·5 million deaths.^2^ Alpha-1 antitrypsin (AAT), a major serine protease inhibitor and anti-inflammatory protein,^3^ may influence the trajectory of SARS-CoV-2 infection. Early in disease, AAT may modulate the severity of SARS-CoV-2 infection via inhibition of TMPRSS-2,^4^ the serine protease that cleaves the SARS-CoV-2 spike protein essential for cellular entry. Later in disease, AAT may inhibit inflammatory proteases, e.g., neutrophil elastases (NE), cysteinyl cathepsins, and metalloproteases downstream to SARS-CoV-2 infection, via direct interactions or via inflammatory cytokines that participate in airway pathologies.^5^ Interleukin-6 (IL-6) has been identified as a pivotal cytokine in SARS-CoV-2 pathogenesis and is associated with the morbidity and mortality of SARS-CoV-2-induced ARDS.^6^^,^^7^ IL-6 is a major inducer of AAT production by the liver, and individuals with SARS-CoV-2 infection who exhibit a blunted AAT response to IL-6 may have a worse prognosis.^6^ IL-6 induction of AAT is mediated largely through the classic pathway whereby IL-6 interacts with the IL-6 receptor (IL-6R) on specific cells.^8^ The IL-6/IL-6R complex then binds to gp130, leading to activation of the JAK/STAT, ERK, and PI3K signal transduction pathways that regulate AAT transcription.

In this study, we investigated the AAT-NE balance in different compartments of the lung and plasma in subjects with SARS-CoV-2 ARDS. The kinetics of IL-6 and AAT in the plasma were measured across the disease course, and the role of plasma IL-6 in induction of hepatic AAT synthesis was explored. We also investigated the effect of IL-6 on beta-galactoside alpha-2-6 sialyltransferase1 (ST6GAL1) expression as this mediates AAT sialylation during acute inflammation, promoting increased binding of AAT to interleukin (IL)-8 and neutrophil activating peptide (NAP)-2, thereby regulating neutrophil chemotaxis.^9^ This was further explored in the present study by isolating and purifying AAT from patients with varying degrees of sialylation and determining the ability of AAT to bind IL-8. The levels and activity of AAT in plasma, and AAT and NE in TAs from the airways of SARS-CoV-2 ARDS were characterized and compared to matched nsARDS controls. The localization of secretory leucoprotease inhibitor (SLPI), the other major airways anti-protease, was evaluated utilizing immunohistochemistry (IHC) of lungs from SARS-CoV-2 and control, non-lung disease patients. IHC studies also investigated neutrophil localization and numbers, AAT and NE in the alveolar compartment of SARS-CoV-2 autopsy lungs. Finally, the effect of IL-6 receptor antagonist, tocilizumab, on AAT levels in SARS-CoV-2-infected patients was characterised *in vivo*.

## Methodology

### Ethics

Ethical approval for patient sampling was in accordance with the Declaration of Helsinki, Good Clinical Practices. Ethical approval for SARS-CoV-2 and nsARDS ICU patient sampling was received from the Beaumont Hospital Ethics Committee (REC #18/52, #17/06 (amended specifically for SARS-CoV-2 ICU subjects)). Further nsARDS patient samples were sourced from the placebo arm of the KARE trial.^10^

Ethical approval for ward level patients treated with tocilizumab was approved by the St. Vincents Healthcare Group Research Ethics Committee.

TA samples from SARS-CoV-2 patients were obtained with informed consent and approval by the University of North Carolina Office of Human Research Ethics, IRB 20-0822).

### Patient selection and sampling

Patients from Beaumont hospital, with a laboratory confirmed SARS-CoV-2 diagnosis (via reverse transcriptase–polymerase chain reaction (RT-PCR) assay of nasopharyngeal swab specimens) requiring ICU admission for intubation and mechanical ventilation for hypoxemic respiratory failure were studied (SARS-CoV-2 ICU, *n* = 20) (Table 1a,1b). Sample size was based on standard cut-offs of 0.05 for type I error and 0.2 for type II error, following observational studies of patients with non-SARS-CoV-2 ARDS.^11^ However, unfamiliarity with the potential breadth of presentations and outcomes in SARS-CoV-2 infection led to our decision to exceed requirements and recruit 20 patients.

**Table 1a:** SARS-CoV-2 ARDS patient demographics for plasma studies.

Demographics of patient cohort	(*n* = 20)
**Age in years**	51.1 +/-13.4
**Male/female**	17/3
**Days since onset of symptoms**	8.7 +/- 3.9
**Symptoms at admission (%)**	
Fever	11 (55)
Dyspnea	13 (65)
Cough	13 (65)
Sputum production	2 (10)
Myalgia	3 (15)
Fatigue	2 (10)
Anorexia	2 (10)
Nausea	3 (15)
Vomiting	1 (5)
Diarrhea	2 (10)
Chest pain	1 (5)
**Circumstances surrounding infection**	
Close contact with infected person	3 (15)
Community-acquired	17 (85)
**Comorbidities**	
Hypertension	11 (55)
Coronary artery disease	1 (5)
Diabetes mellitus	2 (10)
Obesity	11 (55)
**Smoking history**	
Current	2 (10)
Former	1 (5)
Never	14 (70)

**Table 1b:** SARS-CoV-2 ARDS patient clinical status.

Clinical features of patient cohort at time of ICU admission	(*n* = 20)
Temperature >38 °C	9 (45)
Heart rate >100 beats per minute	12 (60)
Respiratory rate >20 breaths per minute	10 (50)
SaO_2_ <80% or requiring FiO_2_ ≥60%	16 (80)
Acute confusion	1 (5)
PaO_2_	9.5 +/- 1.6
Mean arterial pressure	91.6 +/- 16.1
SOFA score	8.05 +/- 2.3
PaO:FiO_2_ on arrival to ICU	160.5 +/- 60.8

Blood samples from the ICU cohort were collected by venipuncture into lithium heparin tubes on admission to ICU and every 2nd day following admission. Plasma was isolated from blood by centrifugation at 350 *xg* for 10 min. nsARDS patients selected for TA samples (*n* = 6, Table 2b) were chosen on the basis of a negative RT-PCR of a nasopharangeal swab and moderate to severe ARDS as per BERLIN criteria, TA samples were collected aseptically, by opening the closed suction device, inserting sterile suction tubing and pulling back by no more than 37 cm to obtain an adequate sample. 8 plasma samples were collected from patients enrolled on the placebo arm of the KARE trial. In ward patients with mild SARS-CoV-2 (*n* = 24, plasma samples were taken pre- and 28 days post tocilizumab infusion. TA samples from SARS-CoV-2 ARDS patients were obtained 1 day post intubation (*n* = 15), 10 from UNC, 5 from Beaumont hospital. All of the TA control group samples from nsARDS patients were also obtained 1 day post intubation (*n* = 6). Plasma was also collected from healthy donors (*n* = 11). All sampled patients were selected at random from a list of medical record numbers, a computer-generated series of random numbers, corresponding to patients with a confirmed infected status. Patients were excluded if they were immunosuppressed, on long-term oral corticosteroids, anti-IL-1, anti-IL-6, or anti-TNF therapy, were pregnant, had active neoplasia, or a history of vasculitis or connective tissue disease. Experiments performed on the samples were undertaken by a blinded investigator who were excluded from the collection of samples and the patients’ clinical care. All experiments involving patient samples were replicated a minimum of 3 times however the total number of samples taken varied depending on potential discharge or death of a patient

### Measurements in plasma

AAT levels in plasma were measured by immunoturbidimetric assay. Plasma IL-6 was measured by Human IL-6 Quantikine ELISA Kit (R&D). Plasma IL-8 was measured by IL-8/CXCL8 DuoSet ELISA Kit (R&D).

### Antibody and reagent validation

All antibodies, reagents and cell lines have been obtained from commercial vendors. Antibodies were used in accordance with the protocols outlined on their corresponding datasheets. Performance for western blotting was validated by inclusion of purified standards of target proteins. Amplicon specificity was verified by amplicon visualisation using agarose gel electrophoresis.

### Western blot analysis of plasma and airway samples

Western blot analysis was performed on patient samples (plasma and TAs) for AAT and SLPI and NE from SARS-CoV-2 ARDS patients (plasma samples *n* = 20, TA samples *n* = 15) along with a control group samples from nsARDS patients (plasma samples *n* = 8, TA samples *n* = 6). Total protein quantities were measured with a BCA Protein Assay Kit (Pierce®). HRP-coupled primary antibodies for AAT (1:2000, GR286990-3, Abcam), NE (1:1000, clone # 950317, MAB91671, R&D systems) and SLPI (1:2000, AF1274, R&D systems, RRID: AB_2302508), were incubated overnight at 4 °C with the target protein, then probed with a secondary HRP-linked antibody (1:1000, 7076S, Cell signalling technology, RRID: AB_330924, and 1:1000, SC2020, Santa Cruz biotechnology, RRID: AB_631728, respectively). Proteins were detected with Immobilon ECL Ultra Western HRP Substrate (Merck Millipore) using a ChemiDoc™ Imaging System (Bio-Rad). Semi-quantification by densitometry was performed with ImageJ software (NIH).

### Co-immunoprecipitation of alpha-1 antitrypsin and IL-8

Binding capacity of IL-8 by AAT was determined by immunoprecipitation of AAT from plasma of healthy donors, or people with SARS-CoV-2 infection prior to and post treatment with tocilizumab, using an anti-AAT antibody (clone 202808, R&D Systems, RRID: AB_2301508) immobilised on amine-reactive beads according to manufacturers’ instructions (Pierce co-immunoprecipitation kit, Thermo). Plasma (20 μl) was diluted 1:5 in Dulbeccos PBS and pre-cleared of non-specifically binding proteins. AAT was then captured overnight at 4 °C with 5 μg antibody per reaction. Bound protein was eluted using 60 μl of elution buffer and assayed by ELISA for AAT or IL-8 at a 1:20 dilution together with flow through fraction and control eluate from beads that lacked antibody.

### AAT and NE activity analysis

AAT-mediated NE inhibition by SARS-CoV-2 plasma (*n* = 20) and NE activity and AAT-mediated NE inhibition in SARS-CoV-2 TAs (*n* = 5) was assessed by fluorescence resonance energy transfer (FRET) analysis as described previously.^12^ AAT was incubated at various concentrations with NE for 20 min at 37 °C and added to a 96 well plate. A working solution of 20 μM of FRET substrate Abz-Ala-Pro-Glu-Glu-Ile-Met-Arg-Arg-Gln-EDDnp was added (3230-v, Peptanova GmbH). Upon addition, the kinetic reaction was immediately measured at 37 °C, over 20 min with readings occurring every 20 s using a Spectra Max M3 microplate reader (Molecular Devices, Berkshire, UK).

NE activity in SARS-CoV-2 TA, and its inhibition by AAT, was measured using a similar protocol. Untreated SARS-CoV-2 TA samples (*n* = 5) and nsARDS TA control group samples (*n* = 5) were added to the plate with and without AAT (Prolastin 1·25 mg/100 µl), a working solution of 20 μM FRET substrate was added, and the kinetic reaction immediately measured at 37 °C, over 20 min with readings occurring every 20 s using a Spectra Max M3 microplate reader.^12^

### Control and SARS-CoV-2 autopsy lungs

Non-disease control human lungs from individuals without a history of pulmonary disease and size mismatch unsuitable for transplantation were provided by the University of North Carolina (UNC) Tissue Procurement and Cell Culture Core (protocol #03-1396) (*n* = 4). SARS-CoV2 infected autopsy lungs (no hospital admission, no intubation) were provided by Drs. Ross. E. Zumwalt (University of New Mexico, Albuquerque, NM), Dr. Alain Borczuk (Weill Cornell Medicine, New York, NY) and Dr. Leigh B. Thorne (University of North Carolina at Chapel Hill, Chapel Hill, NC) (*n* = 8) . Immunohistochemistry was performed according to protocols as previously described.^13^ To probe for specific proteins, primary antibodies for SLPI (1:4000, ab17157, Abcam, RRID:AB_443693), AAT (1:800, A0012, DAKO, RRID: AB_2335672), NE (1:100, MAB91671, R&D systems) and citrullinated histone H3 (1:2000, ab5103, abcam, RRID:AB_304752) were used in 4% normal donkey serum in PBST (phosphate-buffered saline containing 0.1% Triton X-100). Secondary antibodies included biotinylated donkey anti-mouse IgG (1:200); Alexa Fluor 488 donkey anti-rabbit IgG (1:1000, Thermo, RRID: AB_2556546) Alexa Fluor 555 donkey anti-mouse IgG (1:1000, Thermo, RRID: AB_2536180) and Alexa Fluor 647 donkey anti-mouse/rabbit IgG (1:1000). For the immunofluorescence study, the Vector® TrueVIEW Autofluorescence Quenching Kit (Vector laboratories) was used to reduce background staining. Coverslipped slides were scanned using the following settings: Olympus VS200 whole slide scanner microscope with a 60X, 1.4 NA objective, an Olympus confocal microscope with a 20X, 0.75 NA / 60X, 1.4 NA objective, or a Leica Stellaris5 confocal microscope with a 20X, 0.75 NA /63X, 1.4 NA objective.

### AAT and ST6GAL1 mRNA expression

Hep-G2 cells (HB-8085, ATCC, RRID: CVCL_0027) were cultured in Eagles Modified Essential Medium (EMEM, Sigma-Aldrich), supplemented with 10% (w/v) Fetal Calf Serum (FCS, Sigma-Aldrich) and 0·2% (v/v) Primocin (InVivoGen). Hep-G2 cells were stimulated with IL-6 (180 pg/ml) (Abcam) for 6 to 36 h to induce AAT expression. A 12 well plate, containing 2 × 10^5^ cells/well in 1 ml EMEM, was incubated overnight at 37 °C, following which fresh media supplemented with IL-6 (180 pg/ml) +/- tocilizumab (50 μg/ml, Roche) was added. Plasma sourced from patients with SARS-CoV-2 on the first day of their ICU admission was used for stimulation of AAT production in Hep-G2 cells. A 24 well plate format was used, incubating 8 × 10^4^ cells per well in 500 µl of EMEM per well (overnight at 37 °C), following which media was removed and replaced with 250 µl of plasma from six SARS-CoV-2 ICU patients +/- tocilizumab (50 µg/ml) or six nsARDS patients +/- tocilizumab.

After stimulation with either IL-6 or plasma from ICU based SARS-CoV-2 patients/nsARDS patients, Hep-G2 cells were lysed with radioimmunoprecipitation assay buffer (RIPA) (Thermo) containing 1% Triton X-100 and a protease inhibitor cocktail (Sigma-Aldrich) for 2 h at 4 °C, mRNA extracted using the PureLink RNA Mini Kit (Ambion), reverse transcribed using the QuantiTect Reverse Transcription Kit (Qiagen). cDNA was subjected to 45 cycles of amplification for quantitative real-time polymerase chain reaction (qPCR) using the Master I LightCycler® 480 Kit (Roche) and specific primers targeting AAT (forward primer: 5’- ATGCTGCCCAGAAGACAGATA-3’, reverse primer: 5’-CTGAAGGCGAACTCAGCCA-3’) and ST6GAL1 Forward: 5’- AGG TGT GCT GTT GTG TCG-3’ Reverse: 5’- TGT TGG AAG TTG GCT GTG G-3’). Analyses were performed on a LightCycler 480® instrument (Roche) and gene expression calculated with the 2-ΔΔCt method using GAPDH as the reference gene (Forward: 5’- CAT GAG AAG TAT GAC AAC AGC CT -3’, Reverse: 5’- AGT CCT TCC ACG ATA CCA AAG T -3’).

### Isoelectric focusing and immunoblotting

Immunofixation of plasma AAT glycoforms was performed via isoelectric focusing gel electrophoresis (Sebia HYDRASYS) as previously described.^14^ This method was used not only for the verification of AAT phenotypes in SARS-CoV-2 but also to assess glycosylation patterns of AAT in ICU patients and in ward patients receiving standard of care (SOC) +/- tocilizumab matched for similar starting AAT levels and inflammatory profiles. Isoelectric focusing and immunoblotting was also used to determine the difference in AAT glycosylation between the plasma and tracheal aspirates in SARS-CoV-2 ARDS, nsARDS and an anonymised cystic fibrosis patient with known NE in their TA as an inflammatory control.

### AAT sialylation

To analyse AAT sialylation in SARS-CoV-2 subject plasma, AAT was isolated from plasma samples by an Alpha-1 Antitrypsin Select Resin/Tricorn column as previously described.^15^ Isolated samples were chromatographed by fast protein liquid chromatography (AKTA instrument equipped with UV detector and an automatic fraction collector). Samples were denatured by incubation at 100 °C with 1% (w/v) SDS and 1% (w/v) DTT and incubated subsequently overnight with PNGase F [LZ-rPNGaseF-kit] in NP-40 buffered solutions to release N-glycans. The released glycans were labelled with procainamide, labelled samples analysed by by hydrophilic interaction ultra-performance liquid HILIC-UPLC (ACQUITY UPLC®) controlled by Empower software version 3, build 3471), and SARS-CoV-2 sialylation profiles compared to healthy controls (Ludger Ltd. Culham science centre, Oxfordshire, UK).

### Statistical analysis

Statistical analysis was performed in Prism (v9.2, Graphpad). Results were presented as absolute numbers, means ± standard deviation or as percentages as appropriate. Comparisons between two groups with normally distributed data utilized paired t tests. For comparisons of three or more groups, ANOVA was used. Multiple comparisons across multiple groups were by 2-way ANOVA, with *P* values derived by Tukeys’ post-hoc multiple comparison test. A value of *P* < 0·05 was considered statistically significant. Normality of data was tested using both the D'Agostino & Pearson test (sample size allowing) and Shapiro-Wilk test and was confirmed for each group. Multinomial logistic regression (R version 4.1.2) was used to assess the association of the potential disease modifying or confounding factors, age, sex and AAT level at recruitment, with patient cohort and likelihood ratio testing was used to determine interaction between factors.

### Role of funders

Funders had no role in study design, data collection, data analyses, interpretation, or writing of this manuscript.

## Results

### Patient population characteristics

The demographics of the patient populations are shown in Tables 1a–1c, 2a, 2b; control population in supplemental Table 1. Healthy controls were similar in age to patients admitted to ICU with SARS-CoV-2, (49 ± 16 vs 51 ± 13 years) and, though the other cohorts studied were older on average, age was not seen to associate significantly with one cohort over controls (61.3 ± 8 years, *P* = 0.457, and 60.8 years, *P* = 0.285, for nsARDS and SARS-CoV-2 infection requiring non-ICU hospitalisation, respectively). Sex of the patients was not significantly associated with any of the studied conditions.

**Table 1c:** Demographics of ward level patients receiving SOC+/- tocilizumab.

SARS-CoV-2 patients receiving standard of care (SOC) + /- Tocilizumab	SOC	SOC + Tocilizumab
**Number of patients**	6	24
**M/F**	4/2	16/8
**Age**	60.8+/- 11.1	60.8+/-13.6
**SARS-COV-2 Pos+ via nasal swab**	6 (100)	24 (100)
**Mortality Y/N**	0 (0)	2 (12)

**Table 2a:** SARS-CoV-2 ARDS TA sample demographics.

Demographics of ICU TA samples	SARS-CoV-2 TA *n* = 15
Age	60.2+/-13.2
Days since symptom onset to ICU admission	10+/- 7.2
Days since admission until first tracheal aspirate	1
Positive/Negative Respiratory microbiology since ICU admission	7/8
Mortality Y/N	3/12

**Table 2b:** non-SARS-CoV-2 ARDS (nsARDS) TA demographics.

Demographics of ICU TA samples	nsARDS TA *n* = 6
Age	61.3+/-7.9
Days since symptom onset to ICU admission	9+/-6
Days since development of nsARDS until first tracheal aspirate	1
Positive/Negative Respiratory microbiology since ICU admission	0/6
Mortality Y/N	0/6
PaO2:FiO2	156+/-28.7

By comparison with other cohorts of patients with severe SARS-CoV-2 infection requiring intensive care recruited before vaccination availability, our population was of similar age (51 ± 13 vs 58 ± 9 years^16^). Patients had very similar profile with respect to risk factors (BMI > 30: 55% vs 61%; hypertension: 55% vs 48%) and comparable presentation of cough, dyspnea and fever^17^ albeit fewer patients in our study were diabetic (10% vs 24%^16^). Men were somewhat over-represented in our study with respect to ICU SARS-CoV-2 admissions in the study jurisdiction overall (85% vs 69%, *N* = 438^18^) and elsewhere (85% vs 63% male *N* = 41^16^).

The cohort with moderate SARS-CoV-2 infection requiring hospitalisation was well-matched to similar populations elsewhere (61 ± 11 vs 63 ± 16 years, *N* = 80^17^; 63 ± 18 years^19^; 56 ± 14 years^20^). Of the moderate cohort, 66% were male in the present study, which is comparable to studies elsewhere (59%, *N* = 377^20^).

### Plasma AAT levels in SARS-CoV-2 ARDS

**Figure 1:**
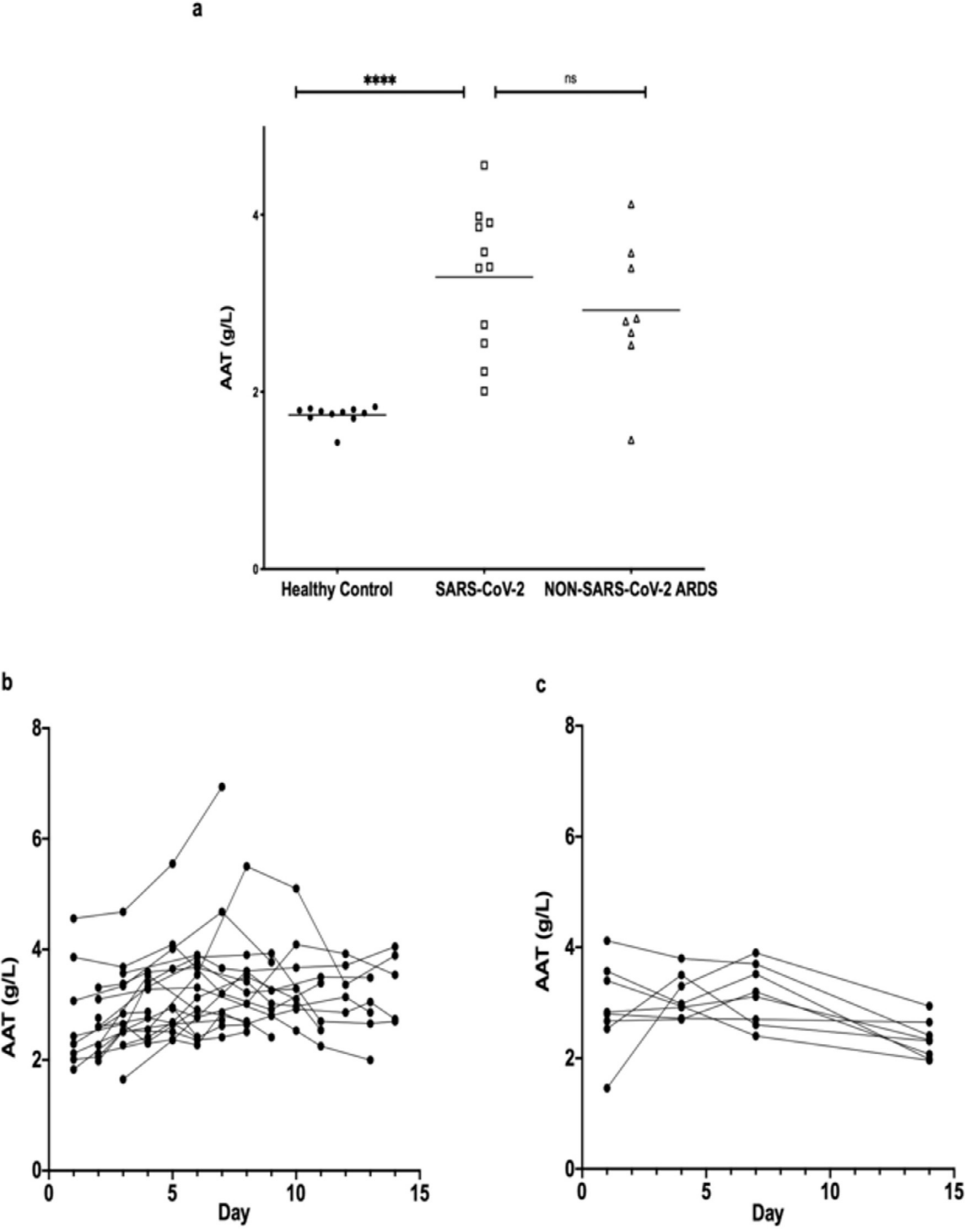
AAT levels in plasma of healthy donors and patients in ICU with SARS-CoV-2 or nsARDS. a. Plasma AAT levels on first day of admission into ICU with SARS-CoV-2 (*n* = 11, 3.294 g/L+/-0.768) or nsARDS (2.923 g/L+/- 0.7984) as compared to healthy controls (*n* = 11) (1.73 g/L+/-0.104), *P* < 0.0001, *P* = 0.0013 respectively with no significant difference between SARS-CoV-2 and nsARDS at day 1. Significance was tested for by one way ANOVA with Tukey multiple comparison test (*p < 0.05). b. Plasma AAT levels in SARS-CoV-2 patients over the first 15 days of ICU admission (Mean: 3.2 g/L +/- 0.7 *n* = 20). c. Plasma alpha-1antitrypsin (AAT) levels in non-SARS-CoV-2 ARDS patients over the first 15 days of ICU admission (2.87 g/L +/-0.607) (*n* = 8).

Plasma AAT levels in SARS-CoV-2 patients on admission to the ICU (3·29 ± 0·8 g/L) were significantly higher than in healthy controls (Mean: 1·74 ± 0·11 g/L, *p* < 0.001, Figure 1a) but similar to nsARDS subjects (Mean: 2·9 ± 0·8 g/L *p* = 0.5273, Figure 1a). The higher levels of AAT in plasma in both ARDS groups persisted throughout their ICU stay (Figure 1b, c). Elevated AAT was prominently associated with each of the conditions, as expected due to its nature as an acute phase protein (SARS-CoV-2 vs healthy controls: log odds 10·93, SE 4·3, *P* = 0.011).

### Form and activity of AAT, NE and SLPI in SARS-CoV-2 plasma and TAs

**Figure 2:**
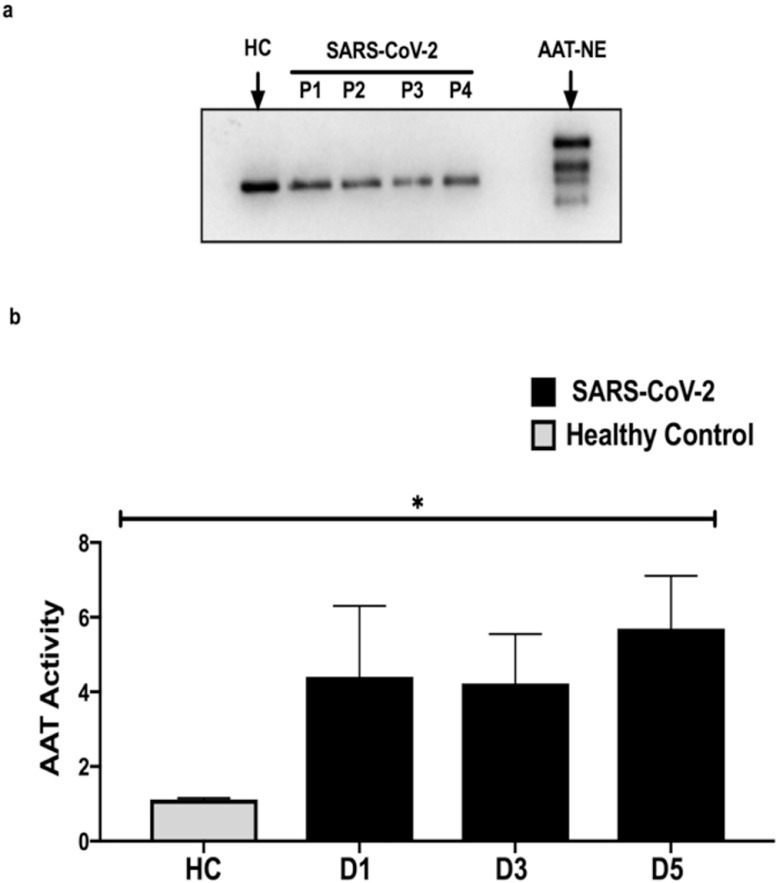
Form and function of AAT in the plasma of SARS-COV-2 ARDS patients. a. Western blot analysis for AAT in SARS-CoV-2 plasma from individual SARS-CoV-2 ARDS patients labelled P1,P2,P3 or P4 shows AAT at the appropriate molecular weight of 52 kDa without AAT-NE complexes or cleavage. b. FRET analysis demonstrate that AAT in the plasma of SARS-COV-2 patients is active and increased compared to healthy controls. *P* < 0.0001. Data represents mean AAT activity as the reciprocal of NE activity +/- SD. Significance was tested for by ANOVA; *p < 0.05.

**Figure 3:**
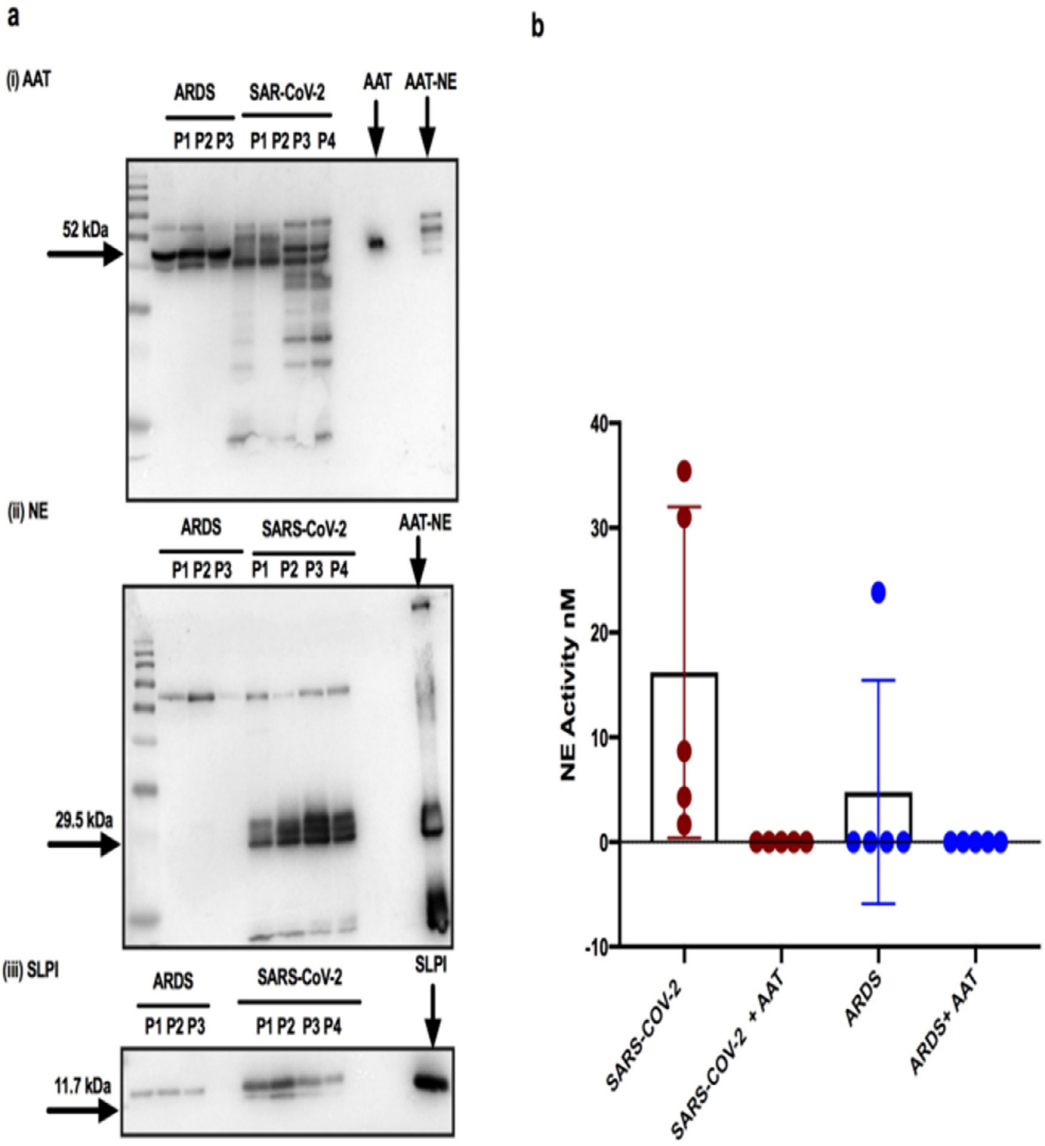
Form and function of AAT, NE and SLPI in plasma and tracheal aspirates (TA) of SARS-COV-2 ARDS patients. a (i) Western blot analysis of AAT in SARS-COV-2 TAs from individual SARS-CoV-2 ARDS patients labelled P1,P2,P3,P4 and the control group of nsARDS patients labelled P1,P2,P3 demonstrates cleavage of AAT and AAT-NE complexes with significantly more cleavage of AAT seen in the SARS-CoV-2 TA samples. (ii, iii) Western blot analysis of NE in the TAs of the same individuals patients show free NE and cleaved SLPI in SARS-CoV-2 TAs in contrast to the nsARDS control group. b. FRET analysis reveals active NE in all 5 TAs from SARS-COV-2 patients on their 1st day of ICU admission and inhibition with exogenous AAT. Only 1 of the 5 nsARDS TA samples demonstrated active NE which was inhibited by exogenous AAT. Significance was tested for by paired T test; *p < 0.05.

Plasma AAT was not complexed or cleaved (Figure 2a) and was fully active against NE (Figure 2b). TA from intubated subjects with SARS-CoV-2 (*n* = 15) and nsARDS (*n* = 6) contained cleaved AAT and AAT-NE complexes on Western analysis (Figure 3a (i)). In the SARS-CoV-2 TA, the AAT was cleaved to a greater extent than the nsARDS TA, and significant free NE was detected by Western blot analyses as well as cleaved SLPI in contrast to nsARDS (Figure 3a (ii) and (iii), respectively). Of all 6 nsARDS TAs only 1 had free NE (Supplemental Figure 1a). As 10 of the SARS-CoV-2 TA samples were in 6M urea, NE activity could not be directly measured as 6M urea interferes with NE activity assays. Five of the SARS-CoV-2 TA samples had no 6M urea added and sufficient volume for FRET protease activity assay. This detected active NE in all five which, was fully inhibited by the addition of plasma purified AAT (Figure 2d).

Commensurate with the Western analysis, active NE was only detected in one of the TA from nsARDS patients. The NE burden in SARS-CoV-2 infected airways, compared to nsARDS, was further demonstrated by iso-electric focusing with immunoblotting for AAT in TAs (supplemental Figure 2a). Glycosylated AAT was present and well defined in the tracheal aspirates of individuals with nsARDS who had no active NE but AAT was significantly degraded in SARS-CoV-2-ARDS TA samples where free NE was detectable.

### IHC analysis of autopsy SARS-CoV-2 lungs

**Figure 4:**
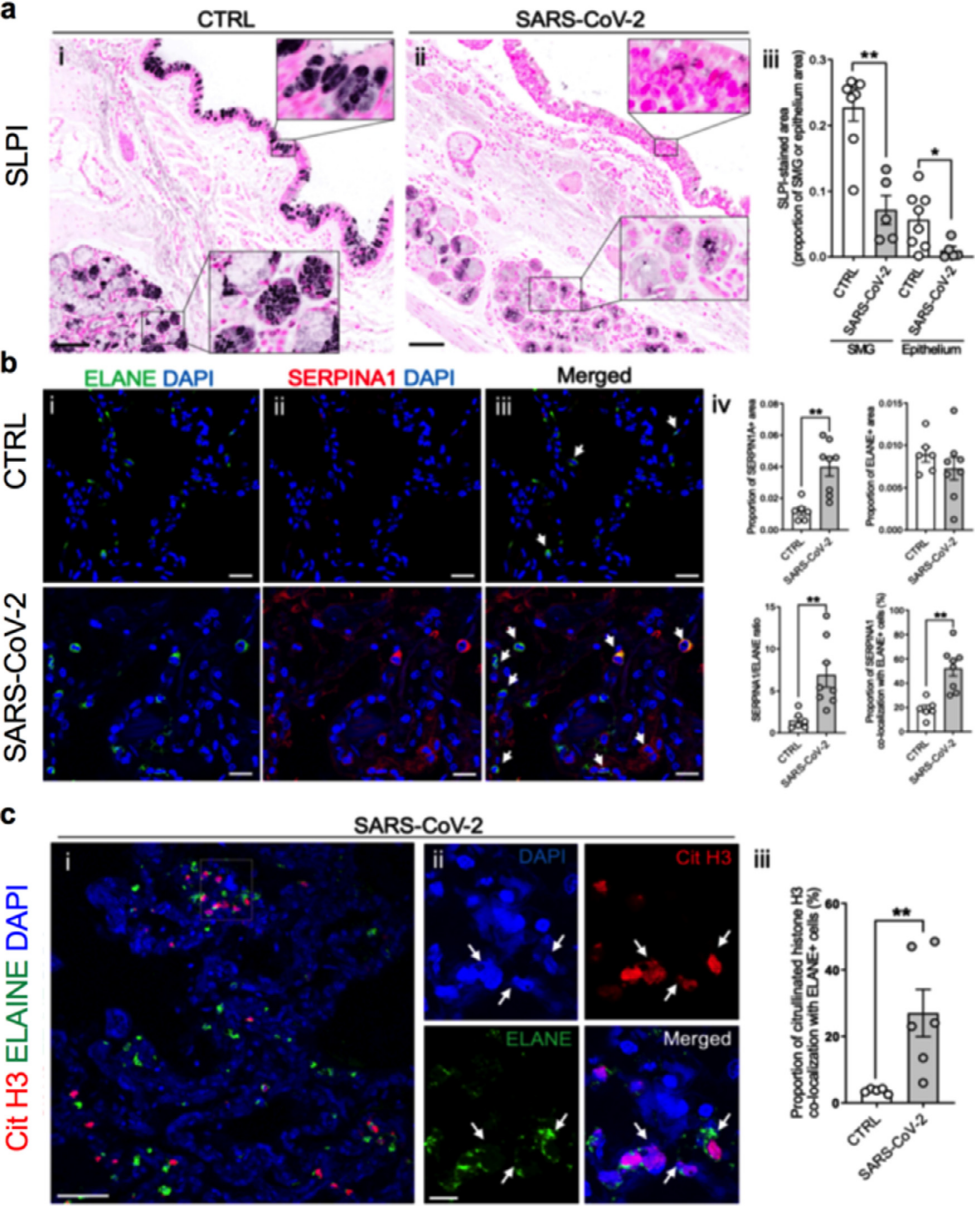
Protein expression in control and SARS-CoV-2 autopsy lungs by immunohistochemistry. a. SLPI protein localization in large airways of control (*n* = 4) (i) and SARS-CoV-2 autopsy lungs (*n* = 8) (ii). Quantification of SLPI protein-stained area in each submucosal grand and epithelium (iii). b. Fluorescent immunohistochemistry of NE (ELANE) and AAT (SERPINA1) in alveoli of control and SARS-CoV-2 autopsy lungs (i–iii). Quantification of each protein-stained area, AAT/NE ratio, and colocalization between AAT and ELANE (iv). c. Fluorescent immunohistochemistry of NE (ELANE) and citrullinated histone H3 (Cit H3) in alveoli of control and SARS-CoV-2 autopsy lungs (i, ii). Arrows showed colocalization of ELANE and Cit H3 (ii). Quantification of co-localization between ELANE and Cit H3 (iii). Histogram bars and error bars represent mean ± SEM. *P < 0.05, **P < 0.01. Scale bars = 100 μm (a); 20 μm (b); 50 μm (ci); 10 μm (Cii). Significance was tested for by Mann-Whitney test (U-test).

Immunohistochemistry (IHC) of control lungs identified SLPI in both superficial epithelia and submucosal glands (SMGs) (Figure 4a). In contrast, in SARS-CoV-2 autopsy lungs, SLPI expression was decreased in both superficial epithelia and SMGs (Figure 4a). Based on previous data demonstrating SARS-CoV-2 infection of superficial airway epithelia, these data are consistent with virus-induced suppression of SLPI expression.^21^

AAT and NE were localized in lungs from 8 subjects who died from SARS-CoV-2 infection. An increase in immuno-detectable AAT was observed in the SARS-CoV-2 autopsy lungs, mainly as free AAT filling alveolar lumens (Figure 4b). There was no increase in neutrophil numbers in the SARS-CoV-2 lungs compared to controls as quantified by ELANE IHC (Figure 4b). AAT appeared by IHC to be in excess of NE in SARS-CoV-2 lungs as indexed by increased AAT/NE (SERPINA1/ELANE) ratios (Figure 4b (iv)) and the increased number of AAT/NE double-positive cells (neutrophils associated with AAT) (Figure 4b (iv)). Notably, a fraction of neutrophils in SARS-CoV-2 autopsy lungs, particularly in the interstitium, were characterized by NET formation as indicated by positive staining for citrullinated histone H3 (Figure 4c, c (iii)).

### AAT and ST6GAL1 response to IL-6 and SARS- CoV-2 ARDS and nsARDS plasma

**Figure 5:**
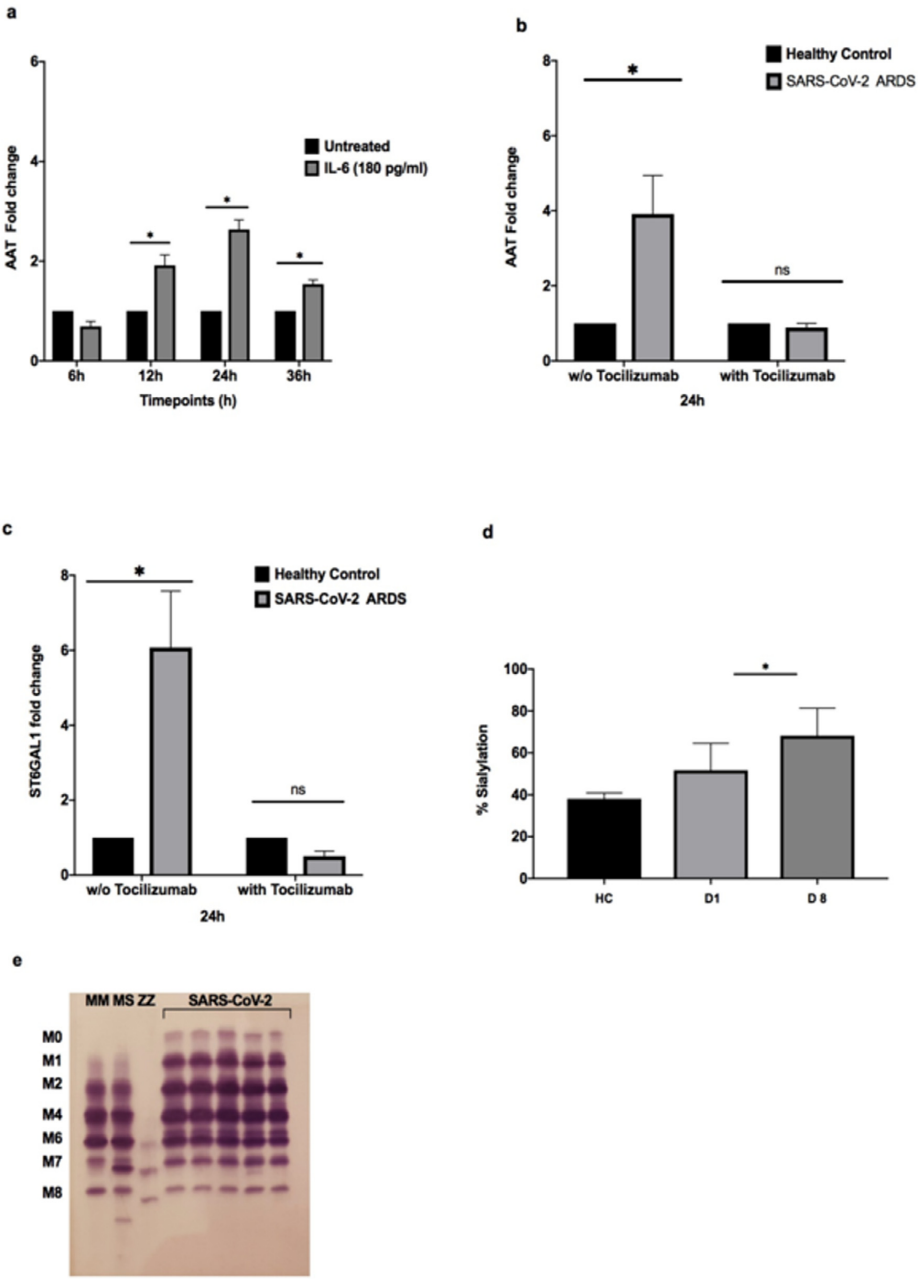
Upregulation of AAT and ST6GAL1 mRNA in response to IL-6 and SARS-CoV-2 ARDS and nsARDS plasma. a. AAT expression in HepG2 cells stimulated with IL-6 over time by RT-qPCR. AAT mRNA production is significantly and consistently raised from 12 h reaching its peak at 24 h. Significance was tested for by two-way ANOVA with Tukey post-hoc test*P < 0.05). b. AAT expression at 24 h in HepG2 cells stimulated with SARS-CoV-2 or nsARDS plasma relative to expression in response to healthy control plasma in the absence (*P < 0.05) or presence of Tocilizumab. c. ST6GAL1 expression in HepG2 cells at 24 h when stimulated with SARS-CoV-2 or nsARDS plasma, relative to expression in response to healthy control plasma in the absence (*P < 0.05) or presence of Tocilizumab. These experiments were repeated three times, with patient plasma being sourced from 20 SARS-CoV-2 patients admitted to ICU. Analyses were performed using a Two-way ANOVA, Tukey's multiple comparisons test. d. Percentage of plasma AAT showing sialylation, from healthy controls compared to SARS-CoV-2 ARDS patients on day 1 (ns,*P* = 0.3) and day 8 of admission to ICU (*P* = 0.02) Analyses were performed using a two-way ANOVA, Tukey's multiple comparisons test. e. Representative isoelectric focusing gel shows presence of highly sialylated M0 and M1 AAT glycoforms in SARS-COV-2 plasma on day 1 post-ICU admission.

AAT expression in hepatic cells, in response to IL-6 administration, at a dose similar to that seen in SARS-CoV-2 ARDS plasma, is delayed, peaking at 24 h after IL-6 administration (Figure 5a). SARS-CoV-2 plasma and nsARDS control plasma also stimulated AAT mRNA production from hepatic cells in a similar fashion (Figure 5b). These responses were largely blocked by tocilizumab, an antibody against the IL-6 receptor (Figure 5b), intimating that IL-6 is the major component of SARS-CoV-2 plasma contributing to AAT upregulation. IL-6 in SARS-CoV-2 and nsARDS control group plasma also induced *ST6GAL1* expression, the enzyme mediating sialylation of AAT (Figure 5c). This was also blocked by tocilizumab, confirming the contribution of IL-6 to sialylation of AAT. The percentage sialylation of AAT in plasma from subjects with severe SARS-CoV-2 infection, collected 8 days into ICU admissions, was increased compared to healthy control plasma (*P* = 0.0211) (Figure 5d). Concurrent with this, there was also an increased abundance of M0 and M1 bands detected in SARS-CoV-2 patients (Figure 5e), and this increase in AAT sialylation glycoforms persisted throughout the ICU stay.

### AAT and IL-6 levels pre-and post-pan-IL-6 receptor antagonist

**Figure 6:**
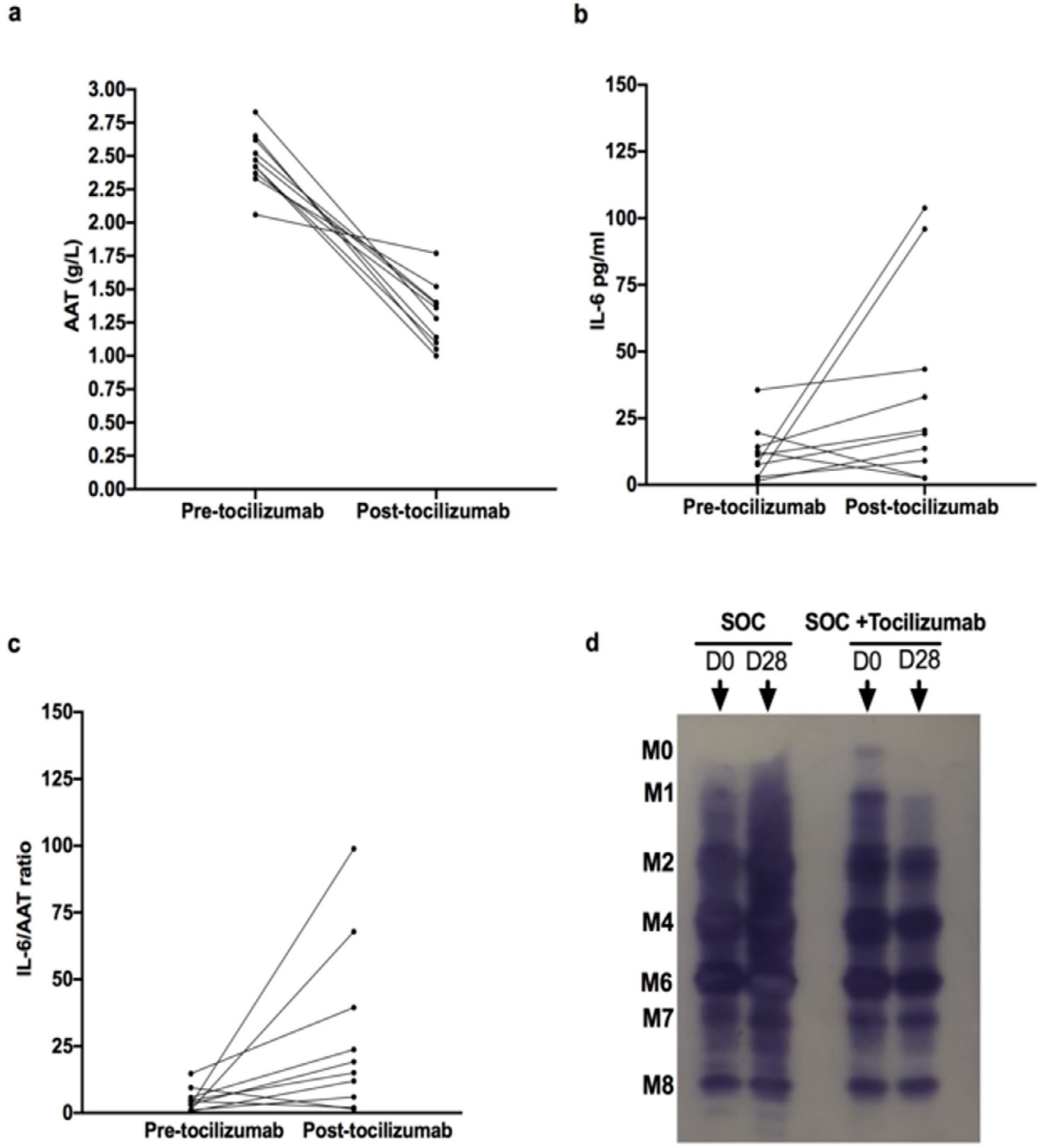
AAT and IL-6 levels pre-and post (28 days) tocilizumab (8mg/kg) in ward level patients with SARS-CoV-2 infection with corresponding isoelectric focussing (IEF) gel. a. Decrease in AAT plasma levels in ward level patients post tocilizumab (*n* = 10) (*P* < 0.0001) (paired T test). b. Maintenance of elevated IL-6 plasma levels in the same cohort of ward level patients throughout their 28 day course (ns=0.099, paired T test). c. Increased IL-6/AAT ratio at day 28 post tocilizumab (*P* = 0.046, paired T test)) d. Glycosylation pattern pre- and post tocilizumab infusion as compared to ward level patients receiving standard of care (SOC), matched for a similar AAT level and inflammatory profile sampled at the same timepoints.

Given the role of IL-6 signalling in regulating AAT secretion, we next examined the effect of tocilizumab on AAT *in vivo*, during acute SARS-CoV-2 infection. Plasma samples were collected from ward level care patients with confirmed SARS-CoV-2 infection pre- and post-tociluzimab infusion. The initial plasma AAT level (2·47 ± 0·2 g/L) was significantly higher than healthy controls (*P* < 0.0001). Patients were dosed with 8 mg/kg tocilizumab (*n* = 24, Table 1c).^22^ A significant decrease in AAT plasma levels was noted 28 days post-tocilizumab (1·3 g/L ± 0·2, *P* < 0.0001) (Figure 6a). In contrast, IL-6 levels remained elevated throughout the 28-day course in these patients (Figure 6b, NS, *P* = 0.0998). These changes were reflected in the increased IL-6/AAT ratio at day 28 post tocilizumab (*P* = 0.046) (Figure 6c), which suggested that IL-6 was no longer regulating AAT production. In ward level COVID-19 patients who received standard of care treatment (SOC) without tocilizumab (*n* = 6), AAT levels remained elevated (2·54 g/L ± 0·4 to 2·27 g/L ± 0·4 at day 28, NS, *P* = 0.1856) (supplemental Figure 3b). Similarly, the IL-6 levels in SOC patients showed no significant decline from their first recorded levels (19·016 pg/ml ± 10·35 pg/ml compared to 15·823 pg/ml ± 13·08; NS, *P* = 0·885, supplemental Figure 3a).

The SOC patients exhibited AAT hyper-glycosylation at the beginning and end of their treatment, denoted by M0 and M1 bands, whereas the tocilizumab treated patients at day 28 showed no M0 or M1 bands despite initially manifesting a hyper-glycosylated AAT profile prior to treatment (Figure 6d). The degree of AAT/IL-8 binding in SARS-CoV-2 samples post tocilizumab infusion was significantly reduced compared to pre-infusion binding (*P* = 0.0011) and in comparison to healthy controls (*P* = 0.0002), reflecting the diminished glycosylation of AAT upon tocilizumab treatment (supplemental Figure 4a).

## Discussion

SARS-CoV-2-infected subjects exhibited evidence of body compartment-specific, protease–antiprotease balances and imbalances. For example, patients with severe SARS-CoV-2 exhibited an acute phase plasma AAT response similar to patients with severe nsARDS (Figure 1a). AAT in SARS-CoV-2 patient plasma was active against NE (Figure 2b) and exhibited the increased sialylation (Figure 5d and e), important for regulating neutrophil chemotaxis.^9^ The increased AAT levels in the plasma of subjects with severe SARS-CoV-2-ARDS or nsARDS was regulated largely by circulating IL-6, as shown by *in vitro* and *in vivo* inhibition with tocilizumab (Figures 5 a,b,c and 6a). *In vitro* data suggested that approximately 24 h is required for IL-6 in plasma to increase AAT levels (Figure 5a), potentially rendering the lung with insufficient anti-protease protection during this time.

While patients with severe SARS-CoV-2 infection exhibited high levels of active AAT, in excess of NE, in their plasma, protease–anti-protease balance in the lung varied with compartment (airways versus alveolus; Figure 4b). In all the TA from SARS-CoV-2-infected subjects, a protease imbalance was observed with AAT mostly cleaved or complexed with NE, and detectable active free NE (Figure 3). On average, these TA were collected 11·1 ± 6·9 days from onset of symptoms and 1 day post ICU admission. In contrast, only one of the patients with nsARDS demonstrated active NE in their TA (airways) which differentiates this syndrome from SARS-CoV-2 ARDS (Figure 3b). This finding suggests that the AAT acute phase response in patients with SARS-CoV-2 ARDS is not sufficient to protect the proximal airways from NE-induced damage. A similar airway protease imbalance has been described in community acquired pneumonia^23^ and cystic fibrosis.^24^

AAT was not the only anti-protease affected by SARS-CoV-2. SLPI, the major locally produced anti-protease in the airways, was cleaved in TAs from SARS-CoV-2 unlike the uncleaved intact form of SLPI seen in nsARDS (Figure 3a). There was also reduced SLPI protein expression in airway epithelia of acutely infected SARS-CoV-2 lungs (Figure 4a). SLPI downregulation has been previously reported with suppression of Nrf2 signalling in SARS-COV-2 infections^25^^,^^26^ and with other viral infections,^27^ and its absence diminishes the airway antiprotease protective shield.^28^ Thus, in subjects with severe SARS-CoV-2 infection, the proximal airway anti-protease and anti-inflammatory protection afforded by locally (airway) produced SLPI, in addition to systemically available AAT, is inadequate to protect against tissue damage. The potential deleterious consequences of this unopposed NE activity include direct tissue damage, impaired ciliary motility, increased mucin secretion, inactivated anti-inflammatory proteins, cleaved complement, complement receptors and immunoglobulins, and initiation of a cycle of inflammation in the lung.^3^ These sequelae are also seen in CF which has led to a series of studies into anti-protease therapy in this condition. Our data suggest this therapeutic option should be considered in SARS-CoV-2 infection. Already a number of small non-randomised controlled trials have suggested some benefit of AAT administration in SARS-CoV-2 infection using both aerosol and intravenous routes of administration.^29^ This raises a number of questions such as which route of administration would be preferable. Certainly in the context of its anti-protease effects and the site of protease-anti-protease imbalance in SARS-CoV-2 ARDS, aerosol delivery of AAT would seem most beneficial but it should also be noted that AAT possesses significant anti-inflammatory effects^3^ and in the context of the severe systemic inflammation seen in SARS-CoV-2 infection an intravenous delivery option would also merit consideration. Another concern is when in the course of the condition such administration should take place and whether it should be given to all SARS-CoV-2 clinical phenotypes. That would require further study specifically to determine whether AAT therapy in SARS-CoV-2 infection mediates its effect mainly through its anti-protease, anti-inflammatory capabilities or through its ability to decrease SARS-CoV-2 entry into cells via its actions on TMPRSS2.

If AAT synthesis and secretion are beneficial responses to SARS-CoV-2 infection, might decreases in AAT synthesis secondary to molecular therapies be harmful? Monoclonal antibodies directed against the IL-6R, e.g., tocilizumab, have been tested in SARS-CoV-2. Reports of tocilizumab efficacy in SARS-CoV-2 have been mixed. A retrospective analysis revealed an association between tocilizumab administration and increased mortality,^30^ ICU admissions, mechanical ventilation and length of stay in subjects with SARS-CoV-2 infection. However, while the recently published results of the RECOVERY and REMAP-CAP trials showed no benefit from tocilizumab alone, they did suggest a benefit from the addition of tocilizumab to high-dose dexamethasone.^31^ Tocilizumab does not discriminate between classical or trans-IL-6 signalling and, therefore, blocks many anti-inflammatory effects of IL-6, including AAT induction. As observed in the present study, the acute phase AAT response may be required to dampen airway inflammation and prevent airway damage during SARS-CoV-2 infections. The noticeable decrease in plasma AAT levels in patients treated with tocilizumab and the coinciding increase in their IL-6 /AAT ratio at day 28 of treatment demonstrates disruption to the normal IL-6-AAT signalling pathway (Figure 6). Not only does this downregulate AAT production *in vivo* but also ST6GAL1, as seen by the resolution of hyperglycoslated M0, M1 AAT bands in patient plasma (Figure 6d). This finding also denotes a reduction in the ability of AAT to bind IL-8, essential for regulating chemotaxis during inflammatory processes, as shown in this study. These data suggest that in future IL-6 blockade trials, special care has to be taken to ensure that inhibition of AAT induction does not worsen an already precarious protease-anti-protease balance in the lungs. In this regard, a more targeted approach, perhaps aimed at IL-6 trans-signalling, using compounds such as soluble gp130, could be considered that would leave the classical IL-6-hepatic AAT synthesis/secretion pathway unaffected or, alternatively, therapies aimed at inhibiting IL-6 should be accompanied by concomitant administration of exogenous AAT.

A limitation of this study is that our patient samples were collected from several different centers. With regards airway samples, the majority of TA samples were from UNC (*n* = 10), where they were treated with 6M urea for safe handling. As a result, FRET analysis of protease activity analysis was not possible in these samples. Five additional TA's were collected from Beaumont hospital which were suitable for NE activity analysis by FRET. No difference was observed on Western analysis of AAT or NE in the UNC 6M urea treated TA samples compared to the TAs from Beaumont hospital, both showing similar patterns of AAT degradation and uncomplexed NE. A suspension of bronchoscopy services during the first wave of the SARS-CoV-2 pandemic prevented us from obtaining small airways lavages. Therefore, the alveolar compartment of SARS-CoV-2 patients was evaluated in UNC by immunohistochemistry of post-mortem specimens. As the vast majority of patients in our ICU at the time had SARS-CoV-2 infection, we had minimal access to nsARDS samples necessitating our using samples from a prior study conducted in a different centre. However these samples were amenable to quanitative and protease activity analysis and thus provided a suitable disease comparison group. Our center was not using tocilizumab as a therapeutic at the time of the study so we accessed patient samples from a sister hospital in Dublin (SVH).

**Figure 7:**
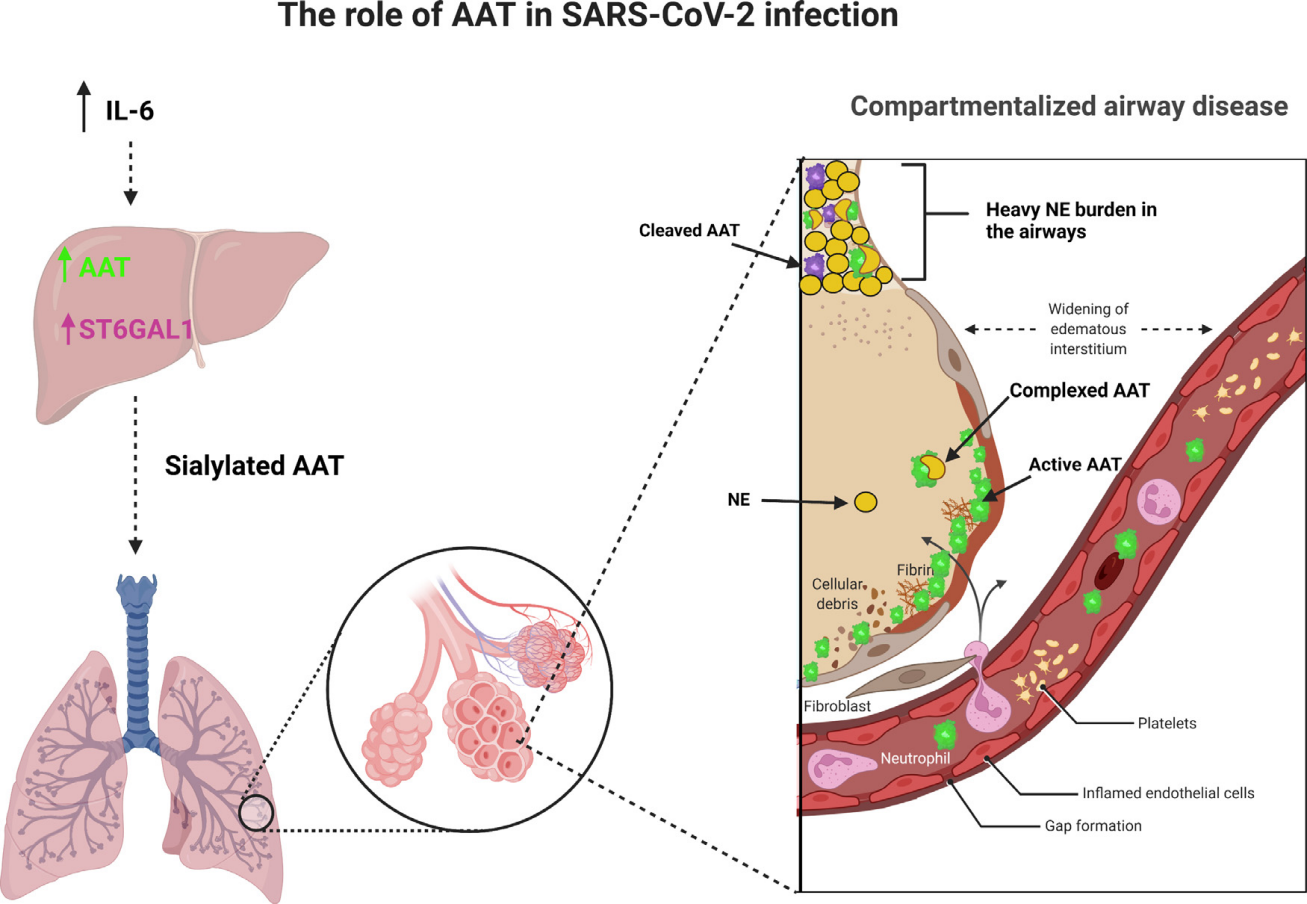
Compartmentalization of protease-anti-protease balance in SARS-CoV-2 ARDS. IL-6 upregulates AAT and ST6GAL1 in the liver resulting in increased secretion of sialylated AAT into the circulation. This AAT crosses into the alveolar compartment where it is able to inhibit NE. However, in the airway compartment the increased NE burden overwhelms the AAT protection leading to unopposed proteolytic action.

In summary, this study demonstrates that AAT responses to SARS-CoV-2 infection are compartmentalized with an appropriate increase in plasma and alveoli but deficient responses in airways (Figure 7). These data suggest administration of intravenous or aerosolized AAT to SARS-CoV-2- infected subject airways may be an appropriate therapeutic option.^32^^,^^33^ Finally, according to these data, caution should be exercised in SARS-CoV-2-infected individuals with respect to administration of IL-6 blocking therapies.

## Declaration of interests

Oisin F McElvaney, Takanori Asakura, Oliver J McElvaney, Suzanne L Meinig, Jose L Torres-Castillo, Robert S Hagan, Claudie Gabillard, Mark P Murphy, Leigh B. Thorne, Alain Borczuk, Emer P Reeves, Ross. E. Zumwalt, Yu Mikami, Tomás Carroll, Kenichi Okuda, Grace Hogan, Jennifer Clarke, Natalie L McEvoy, Ger Curley, Matthew C Wolfgang Cormac McCarthy, Patrick W Mallon and Richard C. Boucher have declared no conflict of interest.

Noel G McElvaney reports grants from PhPharma, Chiesi, consulting fees from Vertex, Takeda, Intellia, non-financial support and fees as a judge for an award from Grifols, stock options from Neuimmune and a patent with Danmarks Tekniske Universitet.

## Appendix Supplementary materials

Image, application 1
