# Supplementary material for: Protease-anti-protease compartmentalization in SARS-CoV-2 ARDS: Therapeutic implications
Source: eBioMedicine. 2022 Feb 22;77:103894. doi: 10.1016/j.ebiom.2022.103894 (PMC8861575; doi:10.1016/j.ebiom.2022.103894)
Supplement: Supplementary file 1 [file mmc1.docx]

**Supplemental Files**

| **File Name** | **Caption** |
| --- | --- |
| EBIOM-D-21-03155R1 supplemental Fig 1 | Supplementary Figure 1 – AAT and NE in nsARDS TA |
| EBIOM-D-21-03155R1 supplemental FIG 2 | Supplementary Figure 2 ‒ IEF of SARS-CoV-2 plasma and TA |
| EBIOM-D-21-03155R1 Supplemental fig 3 | Supplementary Figure 3 ‒ IL-6 and AAT levels of ward level SARS-CoV-2 patients receiving standard of care (SOC) |
| EBIOM-D-21-03155R1 supplemental FIG 4 | Supplementary Figure 4 ‒ AAT/IL-8 ratio of AAT in SARS-CoV-2 or controls |
| EBIOM-D-21-03155R1 Supplemental table 1 | Supplementary Table 1 – Demographic table of health  y controls |

Supplementary Figure 1


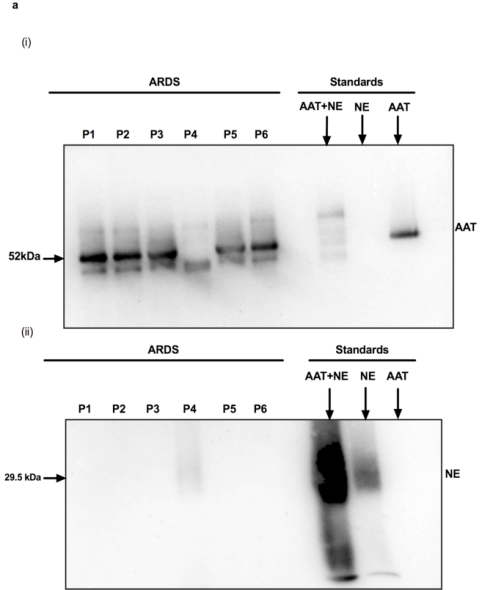


Supplementary Figure 2


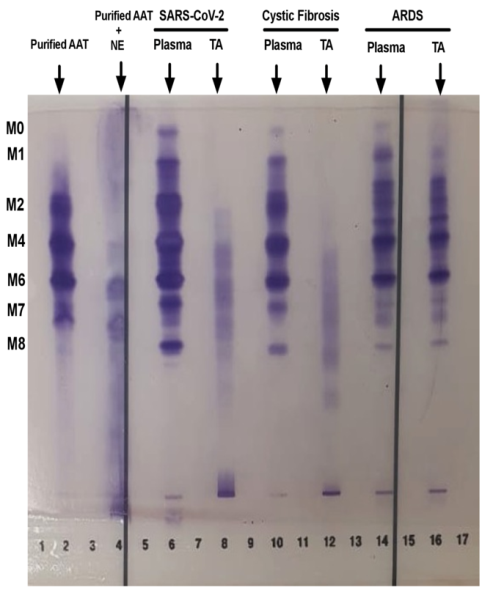


Supplementary Figure 3


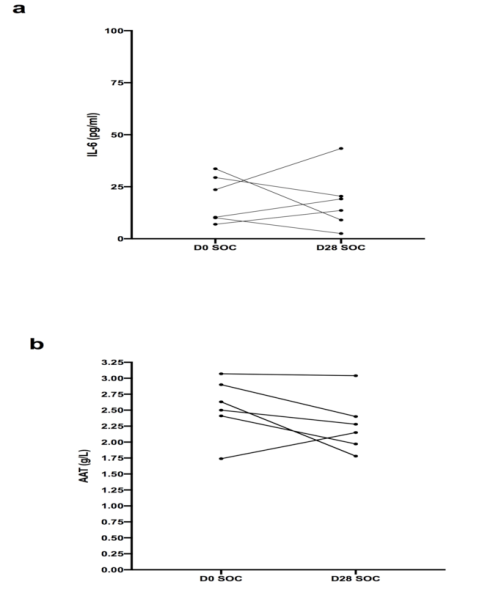


Supplementary Figure 4


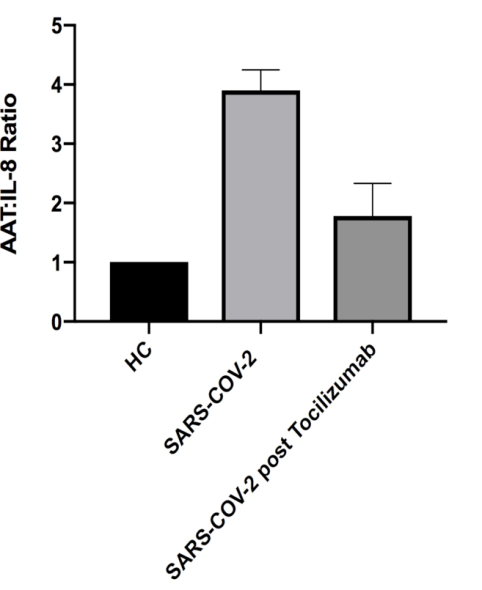


| Demographics of healthy donor cohort | (n=11) |
| --- | --- |
| **Age in years** | 49+/-16.2 |
| **Male/female** | 7/4 |
| **AAT level (g/L)** | 1.73g/L+/-0.104 |
| **IL-6 level (pg/ml)** | 1.67pg/ml+/-1.12 |
| **Any SARS-CoV-2 symptoms at time of sampling (%)** |  |
| Fever | 0 |
| Dyspnea | 0 |
| Cough | 0 |
| Sputum production | 0 |
| Myalgia | 0 |
| Fatigue | 0 |
| Anorexia | 0 |
| Nausea | 0 |
| Vomiting | 0 |
| Diarrhea | 0 |
| Chest pain | 0 |
| **Comorbidities** |  |
| Hypertension | 2 (18) |
| Coronary artery disease | 0 (0) |
| Diabetes mellitus | 1 (0.9) |
| Obesity | 5 (45) |
| **Smoking history** |  |
| Current | 3 (27) |
| Former | 1 (0.9) |
| Never | 7(63.6) |
